# Short Daily Exposure to Environmental Enrichment, Fluoxetine, or Their Combination Reverses Deterioration of the Coat and Anhedonia Behaviors with Differential Effects on Hippocampal Neurogenesis in Chronically Stressed Mice

**DOI:** 10.3390/ijms222010976

**Published:** 2021-10-12

**Authors:** Gerardo Bernabé Ramírez-Rodríguez, Nelly Maritza Vega-Rivera, David Meneses-San Juan, Leonardo Ortiz-López, Erika Montserrat Estrada-Camarena, Mónica Flores-Ramos

**Affiliations:** 1 Laboratorio of Neurogenesis, Subdirección de Investigaciones Clínicas, Instituto Nacional de Psiquiatría “Ramón de la Fuente Muñiz”, Calzada México-Xochimilco 101, Mexico City C.P. 14370, Mexico; meneses.sj.d13@gmail.com (D.M.-S.J.); leosan@imp.edu.mx (L.O.-L.); 2 Laboratorio of Neurpsicofarmacología, Dirección de Neurociencias, Instituto Nacional de Psiquiatría “Ramón de la Fuente Muñiz”, Calzada México-Xochimilco 101, Mexico City C.P. 14370, Mexico; nmvega@imp.edu.mx (N.M.V.-R.); estrada@imp.edu.mx (E.M.E.-C.); 3 Dirección de Enseñanza, Instituto Nacional de Psiquiatría “Ramón de la Fuente Muñiz”, Calzada México-Xochimilco 101, Col. San Lorenzo Huipulco, Tlalpan, Mexico City C.P. 14370, Mexico; monica.flores@imp.edu.mx

**Keywords:** adult neurogenesis, hippocampus, depression, chronic mild stress, environmental enrichment, fluoxetine

## Abstract

Depression is a neuropsychiatric disorder with a high impact on the worldwide population. To overcome depression, antidepressant drugs are the first line of treatment. However, pre-clinical studies have pointed out that antidepressants are not entirely efficacious and that the quality of the living environment after stress cessation may play a relevant role in increasing their efficacy. As it is unknown whether a short daily exposure to environmental enrichment during chronic stress and antidepressant treatment will be more effective than just the pharmacological treatment, this study analyzed the effects of fluoxetine, environmental enrichment, and their combination on depressive-associated behavior. Additionally, we investigated hippocampal neurogenesis in mice exposed to chronic mild stress. Our results indicate that fluoxetine reversed anhedonia. Besides, fluoxetine reversed the decrement of some events of the hippocampal neurogenic process caused by chronic mild stress. Conversely, short daily exposure to environmental enrichment changed the deterioration of the coat and anhedonia. Although, this environmental intervention did not produce significant changes in the neurogenic process affected by chronic mild stress, fluoxetine plus environmental enrichment showed similar effects to those caused by environmental enrichment to reverse depressive-like behaviors. Like fluoxetine, the combination reversed the declining number of Ki67, doublecortin, calretinin cells and mature newborn neurons. Finally, this study suggests that short daily exposure to environmental enrichment improves the effects of fluoxetine to reverse the deterioration of the coat and anhedonia in chronically stressed mice. In addition, the combination of fluoxetine with environmental enrichment produces more significant effects than those caused by fluoxetine alone on some events of the neurogenic process. Thus, environmental enrichment improves the benefits of pharmacological treatment by mechanisms that need to be clarified.

## 1. Introduction

Depression is characterized by neurochemical, neuroplastic, and behavioral alterations [1,2]. As a first-line strategy for treating depression, antidepressant drugs have shown several effects to counteract this neuropsychiatric disorder [3,4,5]. In addition to the neurochemical and behavioral improvement, antidepressant drugs also favor plastic changes in the brain, including but not limited to the generation of new neurons in the dentate gyrus of the hippocampus [6,7,8].

In this regard, several antidepressant drugs such as fluoxetine and citalopram, both acting as selective serotonin reuptake inhibitors (SSRIs), have shown positive effects at the level of the generation of new neurons in the dentate gyrus of the hippocampus [9,10]. Hippocampal new neurons play a role in learning and memory processes and buffering stress exposure [11]. In this sense, it has been shown that the increasing number of new neurons concurs with better behavior in animal models of depression [11]. Additionally, the generation of new neurons is differentially affected along the dorsal–ventral hippocampal subregions by environmental or pharmacological interventions [12]. The relevance of these hippocampal subregions derives from studies that demonstrated the specific participation of the dorsal part in behaviors related to spatial navigation [13], whereas the ventral part of the hippocampus is associated with behaviors related to the mood [14,15].

Interestingly, antidepressant drugs control different events of the neurogenic process in the hippocampus of rodents. Fluoxetine increases cell proliferation, survival, dendrite, and neuronal maturation, as well as the brain-derived neurotrophic factor (BDNF) [9,16,17,18]. Besides, studies performed in post-mortem human brains have shown the effects of antidepressants on some events of hippocampal neurogenesis [4,19]. This evidence has proven the effects of antidepressants as modulators of the neurogenic process or, at least in humans, points in the same direction as studies performed in animal models of depression [4,9,19].

Moreover, there is still controversy about the effects of antidepressant drugs, mainly because their efficacy is variable and incomplete [20]. Thus, it has been proposed that modifications of the living environment may play a role in getting better effects from antidepressant drugs [20]. Recently, fluoxetine’s effects on depression were modified by improving the living environment after cessation of chronic stress in mice [21]. Thus, increasing the quality of life of mice by exposure to a complex environmental enrichment favors neurochemical- and neuroplasticity-related changes [22]. Thus, exposure to various social, cognitive, and physical stimuli acts as a positive factor in treating various behavioral alterations related to chronic stress exposure [23,24]. Among these paradigms, environmental enrichment acts as a potent regulator of the neurogenic process [25]. Environmental enrichment increases cell proliferation, cell survival, dendrite maturation of newborn immature neurons, and newborn cells [12]. In addition, environmental enrichment increases neurotransmitters, growth factors, and neurotrophins, such as BDNF [22]. Interestingly, environmental enrichment improves cognitive functions [26,27] and reverses the decrease of adult hippocampal neurogenesis caused by stress [23].

Based on the above-mentioned evidence, we considered that the inclusion of a short daily exposure to environmental enrichment together with the pharmacological treatment during chronic mild stress could be relevant to increase the efficacy of antidepressant drugs. Although, to the best of our knowledge, this approach has not been analyzed yet. We hypothesized that modifications of the environment through a short daily exposure to environmental enrichment, also known as restricted environmental enrichment, will increase the efficacy of fluoxetine to reverse depressive-like behaviors and hippocampal neurogenesis alterations of mice exposed to chronic mild stress.

## 2. Results

### 2.1. Coat-State and Sucrose Preference after Chronic Mild Stress

**Figure 1:**
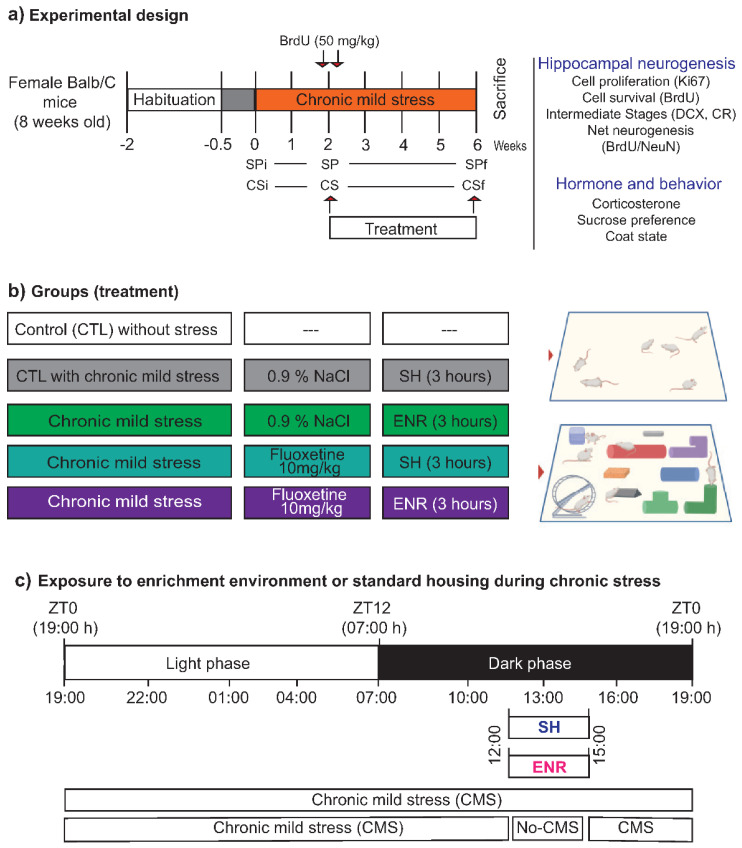
Experimental design. BALB/c female mice were maintained under normal housing until the age of 8 weeks. (**a**) Mice were exposed to chronic mild stress for 6 weeks. After the second week of chronic mild stress, mice were treated with vehicle (CTL), fluoxetine, environmental enrichment (ENR), or the combination of fluoxetine and ENR (**a**,**b**). All drugs or saline were injected at 7:00 h. During the chronic mild stress (CMS) protocol, the coat-state (CS) and sucrose preference (SP) of the BALB/c mice were evaluated initially, during the first week, (CSi or SPi), at one time point during the second week of the CMS protocol (CS or SP), and at a final time point (CSf or SPf). At the beginning of the second week, mice received two injections of BrdU (50 mg/kg; i.p.). (**c**) The exposure time along the day in which environmental enrichment (ENR) or standard housing (SH) were applied are shown. Illustrations were created with BioRender.com.

**Figure 2:**
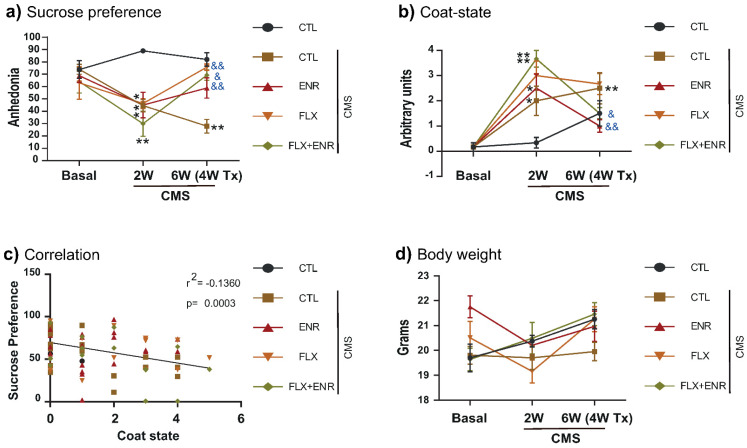
Environmental enrichment and fluoxetine reverse depressive-like behavior. Female BALB/c mice exposed to chronic mild stress (CMS) and treated with fluoxetine (FLX), environmental enrichment (ENR), or the combination (FLX + ENR) were analyzed to determine (**a**) anhedonia and (**b**) the coat state in the second and sixth week of CMS. Data represent the mean ± standard error of the mean (SEM). Two-way ANOVA was followed by the Holm–Sidak test. Symbols “asterisks” in (**a**,**b**) indicate a *p* < 0.001 versus the control un-stressed group. Symbols “&” indicate *p* < 0.033 versus CMS group. (**c**) A significant negative correlation was found between SP and the coat state without significant differences in body weight (**d**).

First, we evaluated the effects of chronic mild stress (CMS) on anhedonia and coat state in mice treated with fluoxetine (FLX), environmental enrichment (ENR), or with the combination FLX+ENR for four weeks (Figure 1 and Figure 2). Two-way repeated-measures analysis of variance (ANOVA) revealed the interaction between treatment (factor A) and time (factor B; F_8,89_ = 5.3, *p* < 0.001 or F_8,89_ = 7.27, *p* < 0.001). Chronic mild stress caused decreased sucrose preference (*p* ≤ 0.010; Figure 2a) and deterioration of the coat (*p* ≤ 0.001; Figure 2b), indicating the presence of behavioral alterations after two weeks of CMS. However, after four weeks of treatment with FLX, environmental enrichment, or their combination in CMS-exposed mice reversed the effects on sucrose preference (*p* ≤ 0.033; Figure 2a) and the deterioration of the coat (*p* ≤ 0.024; Figure 2b). The correlation between sucrose preference and the damage of the coat was significantly negative (Figure 2c), indicating that with the decreased sucrose preference an increased deterioration of the coat occurs (r^2^ = −0.1360, *p* = 0.0003). We did not find differences in the body weight caused by CMS exposure (Figure 2d). The results indicate that FLX, the exposure to environmental enrichment for three hours a day, or their combination during CMS reverts deterioration of the coat and anhedonic behavior of adult female Balb/C mice.

### 2.2. Hippocampal Neurogenic Associated Parameters 

**Figure 3:**
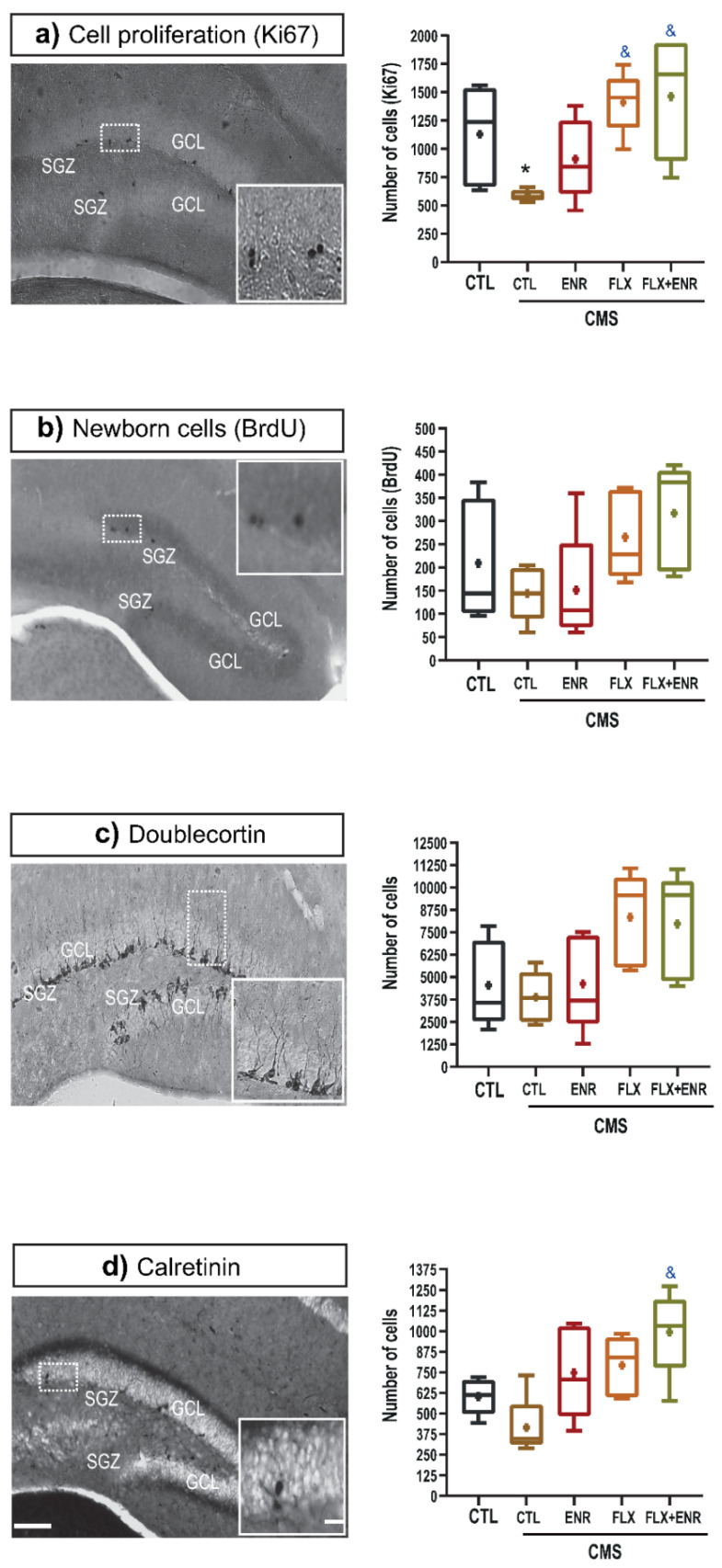
Chronic mild stress (CMS) decreases hippocampal neurogenesis (CTL-CMS) compared with unstressed mice (CTL). Environmental enrichment (ENR), fluoxetine (FLX), or their combination (FLX + ENR) reverses the decreased neurogenesis caused by chronic stress. (**a**–**d**) Representative micrographs of proliferative cells, expressing Ki-67, BrdU, doublecortin or calretinin cells. Micrographs also show the granule cell layer (GCL), molecular layer (ML), and subgranular zone (SGZ). Scale bar = 200 µm. Inserts show amplification. Scale bar = 15 µm. Quantification of Ki-67-labeled cells shows that CMS significantly decreased the number of this cellular population. However, the effects of CMS were reverted by some of the treatments (*n* = 5). Data represent the mean ± standard error of the mean (SEM). Symbols “&” or “*” in (**a**,**d**) indicate *p* ≤ 0.05.

It is known that stress, FLX, and environmental enrichment act as modulators of neuroplastic changes in, but not limited to, the hippocampus (i.e., [9,25]). Thus, we assessed events involved in hippocampal neurogenesis (Figure 3). To determine whether environmental enrichment, FLX, or their combination revert alterations of the neurogenic process in the dentate gyrus of adult female Balb/C mice exposed to CMS, we quantified the absolute number of Ki67- (Figure 3a), BrdU-positive cells (Figure 3b), doublecortin (Figure 3c) and calretinin (Figure 3d) cells. Chronic mild stress significantly decreased the number of Ki67 cells (*p* < 0.05; H = 12.76, d.f. = 4, *p* = 0.013; Figure 3a), but, in the other cellular populations analyzed, CMS caused a decreasing trend in their numbers (Figure 3b,d) without statistical significance. However, FLX or FLX + ENR reverted the decrease of proliferative cells (*p* < 0.05; Figure 3a). A similar increase caused by FLX or FLX + ENR was found in BrdU-, doublecortin-labeled cells (F_4,24_ = 2.39, *p* = 0.08; Figure 3c. H = 9.17, d.f. = 4, *p* = 0.057; Figure 3b,c, respectively) compared with mice exposed to CMS, but without statistical significance. Conversely, the number of calretinin-labeled cells showed a significant increase in rodents exposed to ENR + FLX (*p* = 0.003; Figure 3d) compared to the CMS group.

**Figure 4:**
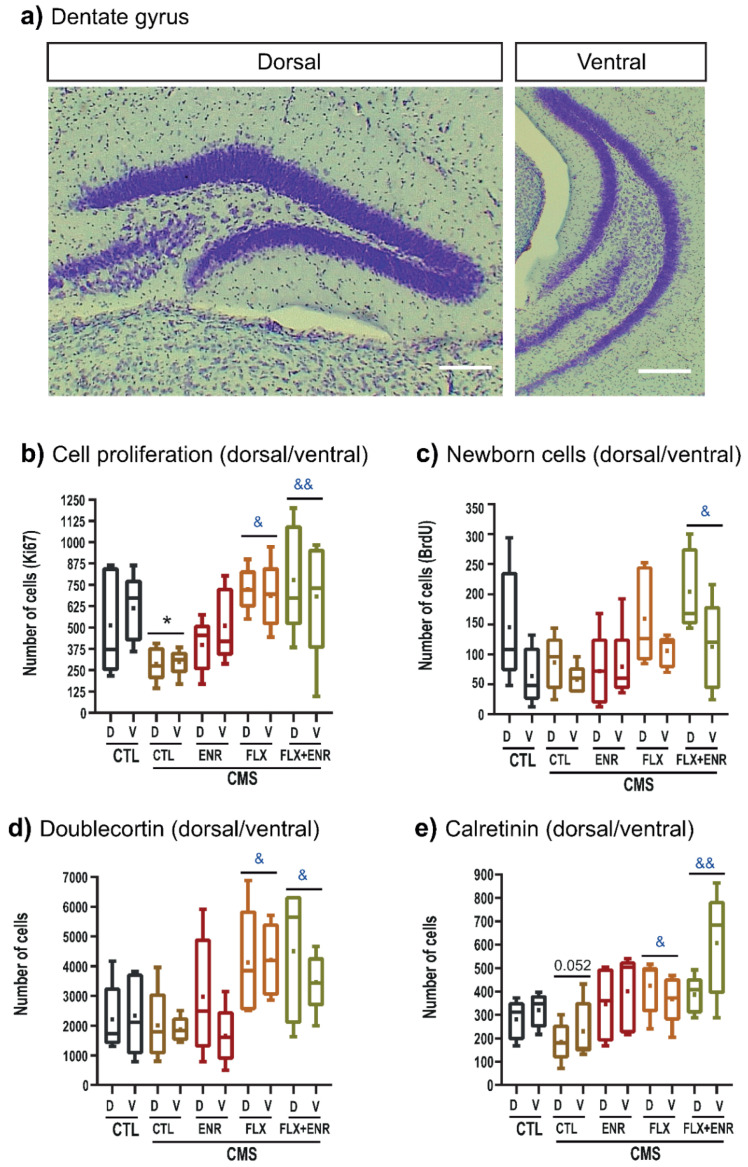
Distribution of cellular populations along the dorsal–ventral hippocampus. (**a**) Representative images after Nissl staining show dorsal (left) or ventral (right) hippocampus. Scale bars represent 100 µm. Chronic mild stress (CMS) decreases hippocampal neurogenesis (CTL-CMS) compared with unstressed mice (CTL). Environmental enrichment (ENR), fluoxetine (FLX), or their combination (FLX + ENR) reverses the decreased neurogenesis caused by chronic stress. (**b**–**e**) Distribution of cellular populations involved in the neurogenic process along the dorsal–ventral (D, V) hippocampus. Similar results were found in Ki67 (**a**), newborn cells (**b**), doublecortin (**c**) and calretinin (**d**) cells. Symbols “&, &&” or “*” in (**a**, **d**) indicate *p* ≤ 0.05.

Furthermore, we analyzed Ki67-, BrdU-, doublecortin, and calretinin-labeled cells in the dorsal and ventral hippocampus (Figure 4a) specifically in the dentate gyrus. Two-way ANOVA for Ki67 cells (Figure 4b) did not show an interaction between treatment (factor A) and region of the hippocampus (dorsal/ventral; factor B; F_4_,_49_ = 0.4, *p* = 0.80). The main effect was produced by treatment (F_4,49_ = 6.53, *p* < 0.001). The chronic mild stress group showed a lower number of proliferative cells than that found in the control unstressed group (*p* = 0.047), but FLX (*p* = 0.002) or its combination with environmental enrichment (*p* < 0.001) reverted the decreasing number of Ki67 cells.

For newborn cells (Figure 4c), two-way ANOVA did not show the interaction between treatment (factor A) and region of the hippocampus (dorsal/ventral; factor B; F_4,49_ = 1.08, *p* = 0.41). However, the main effects were produced by treatment (F_4,49_ = 3.48, *p* = 0.016) and region (F_1,49_ = 7.81, *p* = 0.008). The number of BrdU-positive cells increased in rodents treated with FLX and exposed to ENR (*p* = 0.038) compared to the CMS group. We found also a higher number of BrdU-positive cells in the dentate gyrus in the dorsal than in the ventral hippocampus (*p* = 0.008) in unstressed mice and in mice treated with FLX + ENR.

Two-way ANOVA for doublecortin-labeled cells (Figure 4d) did not show an interaction between treatment (factor A) and region of the hippocampus (dorsal/ventral; factor B; F_4,49_ = 0.54, *p* = 0.70). The main effect was produced by treatment (F_4,49_ = 5.33, *p* = 0.002). Mice treated with FLX (*p* = 0.013) or its combination with ENR (*p* = 0.030) reverted the decreasing number of doublecortin cells observed in the CMS group. We saw similar effects after two-way ANOVA for calretinin cells (Figure 4e; F_4,49_ = 1.50, *p* = 0.21). Again, the main impact was produced by treatment (F_4,49_ = 6.99, *p* < 0.001). Thus, the decreased number in the CMS group (*p* = 0.052) in comparison to the unstressed mice was reverted after treatment with FLX (*p* = 0.023) or its combination with ENR (*p* < 0.001). Thus, FLX and its combination with environmental enrichment reverted neurogenic alterations caused by CMS without a dorsal–ventral hippocampus preference.

### 2.3. Net Neurogenesis after CMS

**Figure 5:**
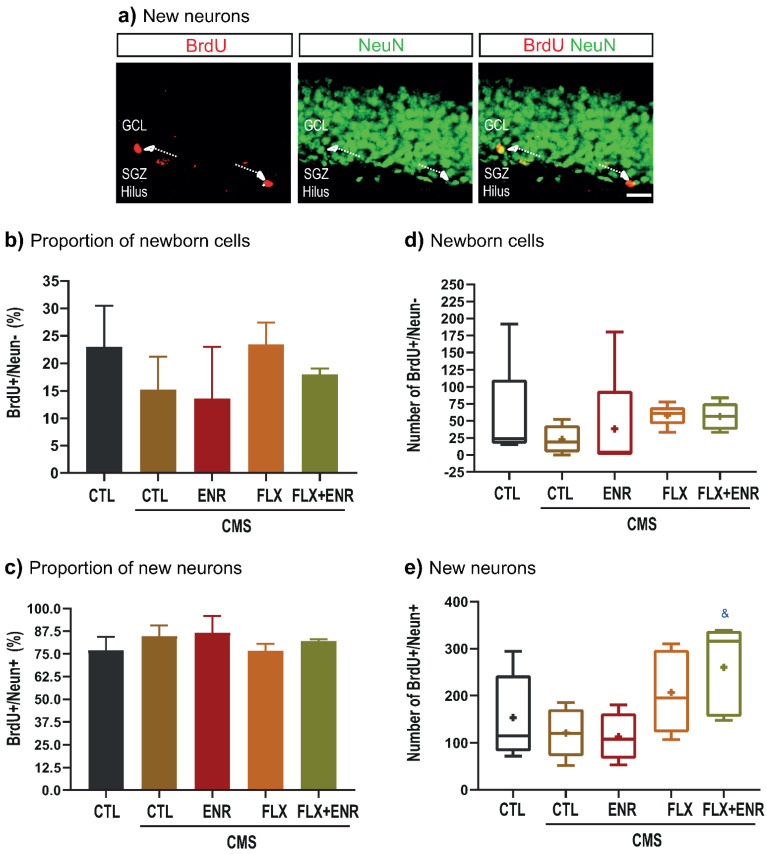
Phenotype of BrdU-positive cells. (**a**) Representative photomicrographs of BrdU-(red) and NeuN-(green) labeled cells. Additionally, the merged picture is shown. Scale bar in a is equal to 30 µm. Proportions of newborn cells (**b**) or new neurons (**c**) are shown without changes among the groups. CTL = unstressed mice, CTL-CMS = stressed mice, ENR = e nvironmental enrichment, FLX = fluoxetine, FLX + ENR = fluoxetine plus environmental enrichment (*n* = 5). Data represent the mean ± standard error of the mean (SEM). Absolute number of newborn cells (**d**) or new neurons (**e**) quantified in all groups exposed or not exposed to CMS. Data are presented as the mean ± standard error of the mean (SEM). *n* = 5. Symbol in (**e**) “&” indicates *p* ≤ 0.05.

The quantitative analysis of newborn neurons (BrdU/NeuN; Figure 5a–c) revealed the same proportion in all groups (F_4,24_ = 0.51, *p* =0.78; Figure 5c). Similarly, net newborn cells were not different among the groups (H = 8.78, d.f. = 4, *p* = 0.06; Figure 5d). However, net neurogenesis (Figure 5e) was increased by FLX plus ENR (*p* = 0.049; F_4,24_ = 3.14, *p* = 0.037; Figure 5e) compared with CMS group.

### 2.4. Correlations of Depressive-like Behavior with Parameters Related to Adult Hippocampal Neurogenesis

**Figure 6:**
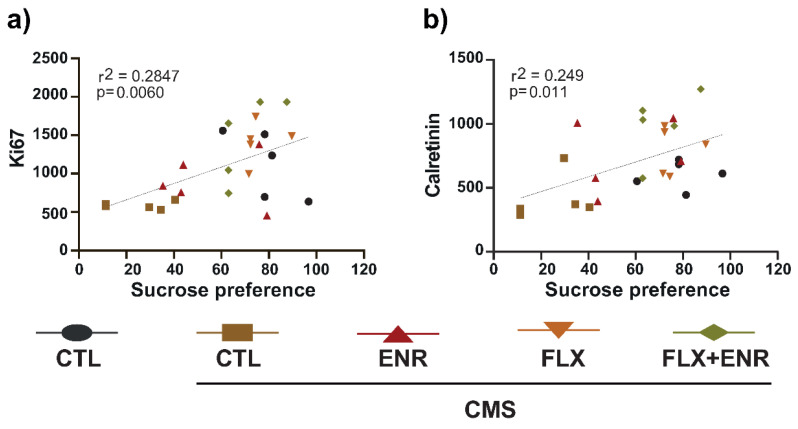
Correlations. (**a**) Scatter graph of Ki67 or (**b**) calretinin with sucrose preference test indicates significant correlation in CTL = unstressed mice, CTL-CMS = stressed mice, ENR = environmental enrichment, FLX = fluoxetine, FLX + ENR = fluoxetine plus environmental enrichment (*n* = 5).

We correlated sucrose preference with the neurogenic parameters evaluated. From the analysis, we only found significant correlation of Ki67- or calretinin-positive cells (Figure 6a, *p* = 0.006, r2 = 0.2847; Figure 6b, *p* = 0.011, r2 = 0.249, respectively) with sucrose preference (Figure 6a,b). However, we did not find a correlation between doublecortin cells, BrdU cells, BrdU+/NeuN+ and sucrose preference nor with coat-state (Figure S1). These results suggest that increased proliferation and calretinin-positive cells correlate with better performance in sucrose preference, suggesting increasing hippocampal neurogenesis with decreased anhedonic behavior in chronically stressed female Balb/C mice.

## 3. Discussion

In this work, we analyzed the contribution of a short daily exposure to environmental enrichment during FLX administration in mice exposed to CMS. We corroborated that FLX reverses CMS effects on depression-associated behavior. We also found that FLX acts as a modulator of some events involved in the hippocampal neurogenic process. However, the short-time exposure to ENR during CMS decreased the deterioration of the coat and reduced significantly anhedonia-related behavior without notorious effects on hippocampal neurogenesis. Likewise, the addition of a short daily exposure to environmental enrichment to FLX showed positive effects on behavior and all parameters of adult hippocampal neurogenesis. Besides, we found a uniform regulation of the neurogenic process along the dorsal–ventral hippocampus in mice treated with FLX or FLX plus ENR.

### 3.1. Behavioral Changes in Response to Chronic Mild Stress and Pharmacological or Environmental Interventions

Chronic mild stress causes behavioral and neuroplastic alterations in rodents [28,29,30,31,32,33,34] and is a relevant factor for depression [20,35,36]. Besides, several strategies have been used to counteract the effects of chronic stress [3,4,5]. Among these strategies, antidepressant drugs reverse behavioral and neuroplastic alterations caused by CMS [37,38]. In this sense, FLX favors several events of neurogenesis occurring in the dentate gyrus of the hippocampus and reverses the depression-related behavioral alterations [39,40,41]. Additionally, constant environmental enrichment that promotes social, physical, and cognitive stimulation can prevent the effects of CMS [30] or reverse the deleterious effects of chronic stress [42]. In this regard, recent studies have pointed out that changing the quality of life will improve the benefits of antidepressant drugs at the behavioral level [20,21]. For example, male C57Bl/6 mice chronically stressed for 14 days followed by the administration of FLX (30 mg/kg) during three weeks in an environmental enrichment paradigm provided in an Intellicage automated system showed a decreased depression-like behavior in terms of anhedonia [21,43]. In these studies, male C57Bl/6 mice were forced to re-establish the social hierarchy as part of the stress protocol. In addition, the paradigm of environmental enrichment included four compartments within four plastic nest boxes and four opaque white boxes and tissue paper [21,43]. Other studies indicated that after cessation of chronic stress, the exposure of rodents to environmental enrichment, as an example of providing life with quality, improves behavior [17,39]. However, those studies [21,43] did not address the effect of an environmental enrichment intervention during the chronic stress period together with the pharmacological treatment. In our study, we found that FLX (10 mg/kg) reverses anhedonia in female Balb/C mice. Similarly, adult male Sprague-Dawley rats exposed to chronic unpredictable stress exhibited depression-like behavior (anhedonia) [44] that reverted after the pharmacological treatment with 10 mg/kg of FLX [44]. Additionally, other authors reported similar results caused by FLX (10 mg/kg) in Balb/C mice [45] and CD-1 mice [46]. Here, our results derived from FLX (10 mg/kg) are in the direction of previous studies reporting the capability of this antidepressant to revert an increased depression-associated behavior like anhedonia. However, we did not find effects of FLX on the coat of female Balb/C mice. In this sense, other studies reported improvement of the deterioration of the coat in male C57Bl/6 mice [47] or male Balb/C mice [45]. It is possible that depending on the sex of mice, it is possible to see the benefits of FLX on both behaviors. However, this speculation requires to be confirmed in an additional study.

Conversely, exposure to environmental enrichment for three hours a day for four weeks in the presence of CMS reverted anhedonia behavior and the deterioration of the coat in female Balb/C mice. Here, the environmental enrichment contained tunnels of different colors and shapes, two-running wheels, pieces of wood, nesting materials, and plastic houses with stairs. In this regard, the occurrence of both behaviors, anhedonia and deterioration of the coat caused by chronic stress, was recently reported in male Balb/C mice [48]. The coat’s physical state, assessing self-grooming behavior, is linked to depression-like behavior in mice [49]. The sucrose preference test allowed assessing anhedonia [50]. Interestingly, our control group showed increased deterioration of the coat that was much lower than that found in chronically stressed mice, but it did not show alterations in anhedonia-related behavior. Similar results on the coat-state in male Balb/C mice not exposed to CMS could be explained by the manipulation of mice during the experiment [51].

Thus, our results point to the direction that exposure to short daily environmental enrichment produces beneficial effects on anhedonia and coat deterioration analyzed during the pharmacological treatment with FLX. Our results with FLX plus ENR are similar to those findings in which adult male Sprague-Dawley rats were treated with FLX or its combination with environmental enrichment, 12 h a day, during three weeks of the pharmacological intervention [44].

The beneficial effects of short times per day of environmental enrichment were initially reported by Rosenzweig [52]. In that study, the exposure for two hours per day for 55 days to an enrichment environment improved behavior related to learning and memory processes [52]. Similar results were obtained in rats after exposure to an enrichment environment for two hours daily for 30 days [53]. A recent study performed with rats with random access to environmental enrichment from 2 to 48 h for 30 days showed better spontaneous activity [54]. Together, these studies support the relevance of limited environmental enrichment exposure to promote behavioral benefits in chronically stressed mice.

Despite the benefits produced by short periods of environmental enrichment, the mechanisms explaining the contribution of short daily exposure to environmental enrichment to the benefits of FLX on behavior are challenging to explain because studies using short exposure to environmental enrichment are scarce. In this sense, FLX treatment in adult male Sprague-Dawley rats exposed to environmental enrichment during chronic unpredictable stress reverted anhedonia and alterations in the expression of proteins located in synapsis, suggesting the importance of synaptic plasticity to amplify the antidepressant effect of FLX [44]. Additionally, exposure to restricted environmental enrichment increased the expression of BDNF and its tropomyosin kinase B receptor in the hippocampus of rats, suggesting that restricted exposure to environmental enrichment boosted the benefits of fluoxetine [54]. However, other aspects should be considered in future studies such as the analysis of neurotransmitters, neurotrophins, or cytokines as possible modulators of the beneficial effects of short daily exposure to environmental enrichment on plasticity against chronic mild stress.

### 3.2. Neurogenic Changes in Response to Chronic Mild Stress and Pharmacological or Environmental Interventions

Adult hippocampal neurogenesis is affected by stress, and some studies have indicated that the newborn neurons are important to buffer stress [11]. Chronic mild stress decreased the number of proliferative cells positive for Ki67 in the subgranular zone of the dentate gyrus [48]. This protein, Ki67, is expressed during all active phases of the cell cycle [55]. In the subgranular zone of the dentate gyrus, its altered expression caused by chronic stress [48] is reverted after treatment with FLX [51,56,57]. Here, we found that CMS caused a strong reduction in Ki67 proliferative cells. Intriguingly, cells derived from asymmetric division in the dentate gyrus, known as the largest fraction of dividing cells, expressed the glucocorticoid receptor [58]. Similarly, neuroblasts positive for doublecortin expressed also the glucocorticoid receptor, suggesting that its expression increased sensitivity to corticosterone [58]. However, cells expressing doublecortin and calretinin were negative for glucocorticoid receptor [58]. Besides, future studies must include the exploration of microglia, which are linked to the effects of CMS [59]. Microglia are important for adult hippocampal neurogenesis. The pro-inflammatory response of microglia affects cell proliferation [60].

FLX or its combination with environmental enrichment reverts decreased proliferation in chronically stressed female Balb/C mice with a uniform distribution along the dorsal and ventral hippocampus. Similar results after treatment with FLX were seen in male Balb/C mice [51], although a higher number of proliferative cells occurred in the ventral hippocampus, but in female Balb/C mice we found a tendency to increased numbers of proliferative cells in the ventral hippocampus of rodents exposed to the short environmental enrichment intervention. These two regions of the hippocampus (dorsal–ventral) are implicated in learning and memory or the modulation of fear and anxiety, respectively [12,13,14,15].

Additionally, we found tendencies to revert the decreasing number of both newborn cells and newborn neurons in the dentate gyrus caused by CMS in rodents treated with FLX. Similar results were previously reported [57]. However, the impact of FLX on the neurogenic process was evident after analyzing the intermediate stages (doublecortin or calretinin expression). Doublecortin is a microtubule-associated protein distributed along the dendrite, and it expresses from the initial steps of neuronal differentiation until more advanced stages of differentiation, including dendritic branching [61,62]: calretinin is a calcium sensor protein during calcium transients occurring with the onset of synaptic activity and linked to the GABA-dependent depolarizing currents [63]. These proteins are temporally expressed during the neurogenic process [64]. Firstly, new immature neurons go through an intermediate phase associated with the expression of doublecortin ranging from progenitor cells to post-mitotic stage, followed by the expression of calretinin together with the first expression of the persistent neuronal marker, NeuN [62,64,65]. Here, FLX showed a tendency to increase the number of doublecortin cells, which reached significance after the analysis along the dorsal/ventral axis. Similarly, the effects of FLX on doublecortin have been previously reported [9]. However, effects of adding environmental enrichment to FLX suggest a significant regulation of all the parameters of the neurogenic process without a preferential regulation of the dorsal–ventral axis, although more cells were found in the ventral region in the case of calretinin.

Interestingly, a positive correlation exists between the increased numbers of proliferative and calretinin cells with better performance in depressive-like behavior. Our results are in line with a previous association of depressive behavior with alterations in the neurogenic process. That study demonstrated that doublecortin cells buffer the effects of stress [11]. In the case of calretinin cells, another study reported their increased number in male C57BL/6 mice treated with FLX (22 mg/kg) [66]. Here, female Balb/C mice treated with FLX (10 mg/kg) or with the combination of FLX plus ENR showed an increased number of calretinin cells, but in mice exposed to FLX plus ENR a tendency to increase the number of calretinin cells in the ventral hippocampus was observed.

Similarly, the administration of ketamine to socially defeated 129S6/SvEv mice produced increasing numbers of calretinin-positive cells in the ventral hippocampus [67]. These data suggest that increased cell proliferation with the consequent increase in the number of calretinin cells may be relevant to mediate the effects of FLX. These effects could be enhanced in the ventral-dentate gyrus by the exposure to a short daily environmental enrichment because mice exposed to environmental enrichment showed tendencies to increase the number of calretinin. However, the absence of a significant effect of a short daily environmental enrichment may also lead us to consider that other neuroplastic mechanisms are involved in the beneficial effects of this intervention combined with FLX. For example, FLX, environmental enrichment, or their combination increased synaptophysin expression and reversed anhedonia suggesting that these interventions may rebuild or repair the functioning of synaptic plasticity seen in unstressed rats [44]. Thus, the evidence suggests that additional mechanisms to regulate the neurogenic process may involve the beneficial effects of a short daily environmental enrichment exposure. Unfortunately, we did not obtain additional samples such as hippocampal protein lysates to evaluate other factors such as neurotransmitters, synaptic-related proteins, or trophic factors, which could help to elucidate how a short daily exposure to environmental enrichment reverted anhedonia and the deterioration of the coat. Additional studies are needed to explore the aspects mentioned above to ascertain the contribution of a short daily ENR exposure to the effects of FLX.

## 4. Materials and Methods

### 4.1. Animals

A total of 30 Balb/C female mice were used in this study. They were held in standard laboratory cages under 12 h light/12 h dark cycles (light phase: ZT0 = 19:00, dark phase: ZT12 = 07:00) at a temperature of 23 ± 1 °C in the animal facilities of the National Institute of Psychiatry “Ramón de la Fuente Muñiz”. Mice had access to food and water ad libitum and were left to acclimatize in their environment until the age of 10 weeks. All institutional and legal regulations regarding animal ethics and handling were followed for in vivo experiments (CEI/C/009/2013). All efforts were made to minimize animal suffering and to reduce the number of animals. In this study, we used females instead of male mice due to the territorial effect present in males. In addition, female mice show less aggression behavior than males [68]. This strain of mice was also chosen due to its sensitivity to stress and its response to chronic antidepressants [45,48].

During the experiment female mice were housed in groups of six rodents per cage (33 × 44 × 20 cm). Unfortunately, we did not check the estrous cycle of mice. Although, some observations done in mice of other projects in our lab suggest that roughly 80–90% of female Balb/C mice were in metestrus phase after chronic mild stress. Interestingly, other studies reported the inhibition of ovulation after stress [69] altering the estrous cycle in rodents [70].

### 4.2. Chronic Mild Stress, Coat-State, and Anhedonia-like Behavior

The CMS was adapted from the original protocol following a recent report [28,29]. Briefly, mice were exposed to a different semi-random type of stressor (Table 1) for six weeks following a previous description (Figure 1a) [30].

To evaluate anhedonia (Figure 1a), mice were deprived of food and water one day every week for 18 h before the sucrose preference test. Then, animals were exposed for one hour to two flasks, one containing 1% sucrose and the other containing drinking water. The concentration chosen has been reported to be more sensitive to stress and less sensitive to motivational manipulations such as food deprivations than the consumption of more concentrated solutions. The exposure to stressors induces a reduction in the consumption of diluted sucrose solutions but increases consumption of sucrose concentrated solutions [71]. To avoid place preference, flask positions were exchanged during every weekly test. Sucrose preference was calculated according to the following formula: percentage of preference = ((sucrose solution intake/total intake) × 100%) as previously reported [40]. Anhedonia was reflected as a reduction in sucrose preference, reaching a value equal to or lower than 50% [30]. We determined sucrose preference during three time points. (1) Before CMS (basal). (2) After the second week of the CMS. Time in which mice showed deterioration of the coat. (3) After the fourth week of treatment with the behavioral or pharmacological interventions. Additionally, the coat state was recorded at the same time points as sucrose preference during the protocol of CMS as an indirect measure of grooming (Figure 1a). According to previous reports, the coat state was evaluated in the following regions of the body: the head, neck, dorsal regions, paws, and tail. According to the physical condition of the coat, a score of 0–1 was assigned to each body area. A score of 0 was assigned to a good state and 1 for an unkempt and dirty coat [31,32]. The score for each animal was the sum of scores from different body parts that were evaluated and divided by the total number of body parts.

### 4.3. BrdU-Labeling, Standard Housing, Environmental Enrichment, and Fluoxetine Administration

Before starting the pharmacological or behavioral interventions or their combination, proliferating cells were labeled with BrdU administered twice, separated by 2 h each (Figure 1a; 50 mg/kg; Sigma, Toluca, Estado de Mexico, Mexico). Mice were distributed into five groups (Figure 1b): (1) mice housed in standard conditions (control, CTL) without CMS; (2) mice exposed to CMS for six weeks (CTL with CMS) that received the vehicle of fluoxetine; (3) mice exposed to environmental enrichment for 3 h/day (ENR) that received the vehicle of fluoxetine; (4) mice treated with 10 mg/kg of fluoxetine (FLX; Abcam, Waltham, MA, USA); (5) mice treated with FLX plus exposure to environmental enrichment (FLX + ENR). The last four groups were maintained in CMS during the pharmacological or environmental interventions (4 weeks of a total of 6 weeks of stress). The vehicle of fluoxetine and fluoxetine were prepared daily (0.9% NaCl; La Pisa, Ciudad de México, Mexico). Both were intraperitoneally injected for four weeks.

Mice were housed in large boxes (34 × 44 × 20 cm), and the environmental enrichment contained tunnels of different colors and shapes, two-running wheels, pieces of wood, nesting materials, and plastic houses with stairs. The complexity was changed every third day to avoid habituation. The exposure to environmental enrichment occurred during the dark phase of the light/dark cycle (Figure 1b,c). The experimental groups that were not exposed to environmental enrichment were housed for 3 h in standard housing conditions (ZT17-ZT20; Figure 1b,c). Six mice per group were used.

### 4.4. Tissue Processing for Immunohistochemistry

Brains were removed, briefly washed three times with phosphate buffer, and fixed for 7 days with 4% *p*-formaldehyde in 0.1 M phosphate buffer (pH 7.4) before stabilization in 30% sucrose in phosphate buffer [21]. Brains were sliced into 40 mm coronal sections on a sliding microtome (Leica, Buffalo Grove, IL, USA). The sections were stored at 4 °C in a cryoprotectant solution before staining according to a free-floating immunohistochemistry method; sections were pre-treated for BrdU-immunodetection by incubation in 2 N HCl for 30 min at 37 °C followed by washes in 0.1 M borate buffer (pH 8.5) for 10 min each [34].

### 4.5. Immunohistochemistry

To analyze the events of the neurogenic process in the hippocampus, we identified key markers of cell proliferation and newborn cells using the peroxidase method, as described previously [34]. Specifically, proliferative cells were identified with a rabbit anti-Ki67 antibody (1:1000; Abcam), newborn cells were identified with a rat anti-BrdU antibody (1:500; Accurate, Westbury, NY, USA), doublecortin cells were identified with a rabbit anti-DCX antibody (1:500; Abcam), and calretinin cells were identified with a mouse anti-calretinin antibody (1:500; Abcam). Secondary biotinylated anti-rabbit, anti-rat, or anti-mouse antibodies were used. (Jackson Immunoresearch, West Grove, PA, USA) [34]. Sections were clarified and mounted in Neo Mount™ reagent (Merck, Kenilworth, NJ, USA).

### 4.6. Quantification of Neurogenic Markers and Phenotypic Analysis

Positive cells for Ki67-, BrdU-, DCX- and calretinin were counted throughout the rostrocaudal extent of the granule cell layer in a series of every sixth section (1-in-6 series) from all animals on a light microscope using a 40× objective (Leica). The number of sections ranged from 10 to 11 per mouse. Counting was conducted as described previously, with a modified optical fractionator method [34]. The cells appearing in the uppermost focal plane were not counted. The estimated total number of labeled cells per granular cell layer was calculated from the estimated resulting numbers multiplied by 6 [34]. The coordinates for the dorsal hippocampus were −1.06 to −2.06 mm relative to bregma; for the ventral hippocampus were −3.08 to −3.80 mm relative to bregma [51].

Phenotypic analysis of newly formed cells in the dentate gyrus was performed in a 1-in-12 series of sections from animals in all groups using double immunofluorescent staining [34]. The co-labeling of BrdU identified new neurons with NeuN. The analysis was performed by confocal microscopy (Zeiss LSM510Meta, Zeiss, Ciudad de Mexico, Mexico) in sequential scanning mode to avoid cross-breeding between channels. Double-labeling was confirmed by three-dimensional reconstructions of z-series covering the entire nucleus (or cell) in question. The primary antibodies were as follows: monoclonal mouse anti-NeuN (1:100; EMD Millipore, Ciudad de Mexico, Mexico) and rat anti-BrdU antibody (1:500; Accurate). Fluorophore-coupled secondary antibodies were as follows: anti-rat TRITC and anti-mouse FITC. All secondary antibodies were raised in donkeys and diluted 1:250 (Jackson Immunoresearch) [30]. Sections were mounted in polyvinyl alcohol with diazabicyclo-octane as an anti-fading agent (PVA-DABCO; Sigma, Toluca, Estado de Mexico, Mexico). The total number per phenotype was calculated from the percentages of every phenotype compared with the total number of BrdU-labeled cells.

### 4.7. Statistical Analysis

The analysis was performed using SigmaPlot 11.0 software (Systat; San Jose, CA, USA). Results are presented as mean ± SEM (standard error of the mean). Sucrose preference, coat state, and the weight along the three time points studied were analyzed by a repeated measures two-way ANOVA, considering the treatment (factor A) and the time (factor B) followed by the Holm–Sidak post hoc test. Some results were also analyzed with a one-way ANOVA followed by the Holm–Sidak post hoc test. However, when the normality test failed, we applied a non-parametric Kruskal–Wallis one-way ANOVA on ranks followed by the multiple comparison procedures within the Dunn’s method. Comparison between treatment (factor A) and region of the dentate gyrus (dorsal or ventral; factor B) was performed with a two-way ANOVA followed by the Holm–Sidak post hoc test. Box plots indicate the mean (Croix with the box) for each experimental condition. The filled lines indicate the median on the 25th and 75th percentiles in the box and the minimum and maximum in whiskers. Differences were considered statistically significant at *p* ≤ 0.05. The correlation between two variables or per group was analyzed with the Pearson correlation coefficient using the SigmaPlot 11.0 software. 

## 5. Conclusions

The association of adult hippocampal neurogenesis with depression has been supported in preclinical and post-mortem reports, suggesting that new neurons buffer the effects of stress. However, the efficacy of antidepressant drugs is variable and incomplete, pointing out the relevance of quality of life to improve their benefits. Thus, short interventions of enriched environments could help to increase antidepressant efficacy and could induce modifications of other aspects related to neuroplasticity. Albeit environmental enrichment per se did not modify net neurogenesis, data of its combination with FLX suggest that modifying the environment improves the effects of the antidepressant drug. Despite the beneficial effects of environmental enrichment to prevent or revert depression-associated behavior, more studies are needed to establish the required environmental enrichment exposure time to counteract CMS effects, increase the efficacy of antidepressants, and know the mechanisms through which the environmental intervention acts. The impact of environmental enrichment to improve the efficacy of pharmacological treatment to revert CMS effects warrants additional studies.

## Figures and Tables

**Table 1:** Stressor used in the chronic mild stress paradigm.

Stressors and Exposure Time	
Grouping housing (6 mice per cage)	8 h
Water deprivation	18 h
Food deprivation	18 h
Continous light	24 h
Cold room (4 °C)	15 min
Stroboscopic light	3 h
Constant motion (100 rpm)	30 min
White noise	12 h
Movement restriction	1 h
Dirty or wet cage	12 h

During the chronic mild stress protocol two or three stressors were applied daily. The sequence and combination were changed every week. The duration of each stressor is shown in the right column of the table.

## Data Availability

All relevant data are within the article.

## Supplementary Materials

The following are available online at https://www.mdpi.com/article/10.3390/ijms222010976/s1.

## Abbreviations

SSRIs	Selective serotonin reuptake inhibitors
BDNF	Brain-derived neurotrophic factor
CMS	Chronic mild stress
FLX	Fluoxetine
ENR	Environmental enrichment
